# ﻿A new *Arthrinium*-like genus of Amphisphaeriales in China

**DOI:** 10.3897/mycokeys.92.86521

**Published:** 2022-08-01

**Authors:** Ning Jiang, Hermann Voglmayr, Chun-Yan Ma, Han Xue, Chun-Gen Piao, Yong Li

**Affiliations:** 1 Key Laboratory of Biodiversity Protection of National Forestry and Grassland Administration, Ecology and Nature Conservation Institute, Chinese Academy of Forestry, Beijing 100091, China Ecology and Nature Conservation Institute, Chinese Academy of Forestry Beijing China; 2 Department of Botany and Biodiversity Research, University of Vienna, Rennweg 14, A-1030 Vienna, Austria University of Vienna Vienna Austria; 3 Natural Resources and Planning Bureau of Rizhao City, Rizhao 276827, China Natural Resources and Planning Bureau of Rizhao City Rizhao China

**Keywords:** *Apiospora*, *Arthrinium*, *Neoarthrinium*, phylogeny, taxonomy

## Abstract

Species of *Arthriniums. l.* are usually known as endophytes, pathogens or saprobes occurring on various hosts and substrates and are characterised by globose to subglobose, sometimes irregular, dark brown and smooth-walled or finely verruculose conidia, always with a truncate basal scar. Currently, *Arthriniums. l.* contains two phylogenetically distinct clades, namely, *Apiospora* and *Arthriniums. s.* However, *Arthriniumtrachycarpi* and *Ar.urticae* have still not been properly classified. With new isolates from diseased leaves of *Lithocarpusglaber* collected in China, we propose the new *Arthrinium*-like genus *Neoarthrinium* in Amphisphaeriales. Based on the morphology and phylogeny of multiple loci, the new genus is established with the type species, *N.lithocarpicola* and three new combinations, *N.moseri* (syn. *Wardomycesmoseri*), *N.trachycarpi* (syn. *Ar.trachycarpi*) and *N.urticae* (syn. *Ar.urticae*) are added to this genus.

## ﻿Introduction

Apiosporaceae, including *Arthrinium*-like taxa, was proposed to accommodate genera with apiosporous hyaline ascospores and a basauxic, *Arthrinium*-like conidiogenesis (Hyde et al. 1998). In a recent outline of Sordariomycetes, Hyde et al. (2020) accepted five genera viz.Appendicospora, *Arthrinium*, *Dictyoarthrinium*, *Endocalyx* and *Nigrospora* in family Apiosporaceae. Soon thereafter, *Dictyoarthrinium* was transferred to Didymosphaeriaceae, based on a multigene phylogeny (Samarakoon et al. 2020). Subsequently, Pintos and Alvarado (2021) separated *Apiospora* from *Arthrinium*, based on the study of the type species of both genera and on multigene phylogeny. Recently, Konta et al. (2021) transferred *Endocalyx* toCainiaceae, based on morphological and phylogenetic evidence and Samarakoon et al. (2022) described the new family Appendicosporaceae forAppendicospora. Therefore, Apiosporaceae currently contains three genera, viz. *Apiospora*, *Arthrinium* and *Nigrospora*.

Until the study of Pintos and Alvarado (2021), the genera *Apiospora* and *Arthrinium* were considered synonymous, the first being used for the sexual morph and the second for the asexual morph in dual nomenclature (Réblová et al. 2016). Following the abandonment of dual nomenclature, the older name *Arthrinium* was recommended for use in unitary nomenclature (Réblová et al. 2016). The genus *Arthrinium* was proposed by Kunze and Schmidt (1817) and validated by Fries (1832) with *Ar.caricicola* as the generic type. *Apiospora*, the type genus of Apiosporaceae, was typified with *Ap.montagnei*, a new name for *Sphaeriaapiospora* (Saccardo 1875). However, the phylogenetic identity of *Ap.montagnei* has been confused because multiple names in *Arthrinium* have similar sexual morphs that have been referred to as *Apiosporamontagnei* (Hudson et al. 1976; Pintos et al. 2019; Pintos and Alvarado 2021). New collections from the original region and hosts (*Arundo*, *Piptatherum*) of *Ap.montagnei* have been isolated in pure culture and sequenced (Crous and Groenewald 2013; Pintos et al. 2019). Five species, previously placed in *Arthrinium*, are classified in *Apiospora*. Two of these phylogenetically distinct species, *Ap.marii* and *Ap.phragmitis*, are morphologically similar to *Ap.montagnei* (Pintos and Alvarado 2021), but due to a lack of sequence data from the type, it cannot be determined which of these two species should become a synonym of *Ap.montagnei*. Irrespective of these taxonomic uncertainties in species concept, recent multigene phylogenies revealed that *Arthrinium* and *Apiospora* represent two well-supported, distinct lineages close to *Nigrospora* in Apiosporaceae (Pintos and Alvarado 2021; Samarakoon et al. 2022). However, two *Arthrinium* species resembling *Apiospora* in conidial morphology, viz. *Ar.trachycarpi* and *Ar.urticae*, were not considered in these studies.

*Arthrinium*-like species are globally distributed, inhabiting various substrates, mainly associated with plant tissues as endophytes, pathogens and saprobes (Cooke 1954; Minter 1985; Larrondo and Calvo 1992; Senanayake et al. 2020; Feng et al. 2021; Jiang and Tian 2021; Tian et al. 2021). Some species are important plant pathogens; for example, *Ap.arundinis* causes bamboo brown culm streak, chestnut leaf spot and barley kernel blight (Martínez-Cano et al. 1992; Chen et al. 2014; Jiang et al. 2021), while *Ap.sacchari* causes damping-off of durum wheat (Mavragani et al. 2007). Another species, *Ar.phaeospermum*, can cause dermatomycosis in humans (Zhao et al. 1990).

In the present study, new *Arthrinium*-like isolates were collected and morphologically examined and their phylogenetic affiliation was determined by analyses of a combined matrix of ITS, LSU, *tef1* and *tub2* sequences. The aim of this study was to determine the phylogenetic placement of *Ar.trachycarpi*, *Ar.urticae* and our new isolates within Amphisphaeriales, which resulted in the identification of a new phylogenetic lineage with isolates belonging to neither *Arthrinium* nor *Apiospora*. As a result, a new genus is established for these isolates.

## ﻿Materials and methods

### ﻿Isolation and morphology

Diseased leaves of *Lithocarpusglaber* were observed and collected in Guangdong Province of China (39 m elevation; 23°8'52"N, 113°27'18"E), packed in paper bags and transferred to the laboratory for pure culture isolation. The samples were first surface-sterilised for 1 min in 75% ethanol, 3 min in 1.25% sodium hypochlorite and 1 min in 75% ethanol, rinsed for 2 min in distilled water and blotted on dry sterile filter paper. Then, the diseased areas of the leaves were cut into 0.5 × 0.5 cm pieces using an aseptic razor blade, transferred on to the surface of potato dextrose agar plates (PDA; 200 g potatoes, 20 g dextrose, 20 g agar per litre) and incubated at 25 °C to obtain pure cultures. The cultures were deposited in the China Forestry Culture Collection Center (CFCC; http://cfcc.caf.ac.cn/) and the specimen was deposited in the Herbarium of the Chinese Academy of Forestry (CAF; http://museum.caf.ac.cn/).

The morphology of the isolates was studied, based on sporulating axenic cultures grown on PDA in the dark at 25 °C. The conidiomata were observed and photographed under a dissecting microscope (M205 C, Leica, Wetzlar, Germany). The conidiogenous cells and conidia were immersed in tap water and then the microscopic photographs were captured with an Axio Imager 2 microscope (Zeiss, Oberkochen, Germany), equipped with an Axiocam 506 colour camera using differential interference contrast (DIC) illumination. For measurements, 50 conidiogenous cells and conidia were randomly selected. Culture characteristics were recorded from PDA after 10 d of incubation at 25 °C in the dark.

### ﻿DNA extraction, PCR amplification and phylogenetic analyses

Genomic DNA was extracted from colonies grown on cellophane-covered PDA using a cetyltrimethylammonium bromide (CTAB) method (Doyle and Doyle 1990). DNA was checked by electrophoresis in a 1% agarose gel and the quality and quantity were measured using a NanoDrop 2000 (Thermo Scientific, Waltham, MA, USA). The following primer pairs were used for amplification of the gene regions sequenced in the present study: ITS1/ITS4 for the ITS1-5.8S-ITS2 nrDNA region (ITS) (White et al. 1990); LR0R/LR5 for the 28S nrDNA region (LSU) (Vilgalys and Hester 1990); EF1-728F/EF2 for the translation elongation factor 1-α (*tef1*) gene (O’Donnell and Cigelnik 1997; Carbone and Kohn 1999); Bt2a/Bt2b for the beta-tubulin (*tub2*) gene (Glass and Donaldson 1995). The PCR conditions were set as follows: an initial denaturation step of 5 min at 94 °C, followed by 35 cycles of 30 s at 94 °C, 50 s at 52 °C (ITS and LSU) or 54 °C (*tef1* and *tub2*) and 1 min at 72 °C and a final elongation step of 7 min at 72 °C. The PCR products were assayed via electrophoresis in 2% agarose gels. DNA sequencing was performed using an ABI PRISM 3730XL DNA Analyser with a BigDye Terminator Kit v.3.1 (Invitrogen, USA) at the Shanghai Invitrogen Biological Technology Company Limited (Beijing, China).

The quality of the chromatograms obtained was checked and the nucleotide sequences were assembled using SeqMan v.7.1.0, the DNASTAR lasergene core suite software (DNASTAR Inc, Madison, WI, USA). Reference sequences were retrieved from the National Center for Biotechnology Information (NCBI; https://www.ncbi.nlm.nih.gov), based on related publications (Crous and Groenewald 2013; Wang et al. 2018; Liu et al. 2019; Pintos and Alvarado 2021; Samarakoon et al. 2022). Sequences were aligned using MAFFT v. 6 (Katoh and Toh 2010) and corrected manually using MEGA 7.0.21 (Kumar et al. 2016).

The phylogenetic analyses of the combined loci were performed using Maximum Likelihood (ML) and Bayesian Inference (BI) methods. The ML was implemented on the CIPRES Science Gateway portal (https://www.phylo.org) using RAxML-HPC BlackBox 8.2.10 (Stamatakis 2014), employing a GTRGAMMA substitution model with 1000 bootstrap replicates. The Bayesian posterior probabilities (BPP) were determined by Markov Chain Monte Carlo (MCMC) sampling in MrBayes v. 3.2.6 (Ronquist et al. 2012). The six simultaneous Markov chains were run for 1 M generations, starting from random trees and sampling trees every 100^th^ generation and 25% of aging samples were discarded, running until the average standard deviation of the split frequencies dropped below 0.01. The phylogram was visualised in FigTree v.1.3.1 (http://tree.bio.ed.ac.uk/software) and edited in Adobe Illustrator CS5 (Adobe Systems Inc., USA). The newly-generated nucleotide sequences were deposited in GenBank (Table 1).

**Table 1.** Isolates and GenBank accession numbers used in the phylogenetic analyses.

Species	Strain	Host	Origin	GenBank accession numbers			
				ITS	LSU	*tub2*	*tef1*
*Allelochaetaacuta*	CPC 16629	*Eucalyptusdives*	Australia	MH554086	MH554297	MH554758	MH554519
*Allelochaetaneoacuta*	CBS 115131	*Eucalyptussmithii*	South Africa	JN871200	JN871209	MH704627	MH704602
*Amphisphaeriamicheliae*	MFLUCC 20-0121	*Micheliaalba*	China	MT756626	MT756620	MT774371	NA
*Apiosporaacutiapica*	KUMCC 20-0209	*Bambusabambos*	China	MT946342	MT946338	MT947365	MT947359
*Apiosporaacutiapica*	KUMCC 20-0210	*Bambusabambos*	China	MT946343	MT946339	MT947366	MT947360
*Apiosporaarundinis*	CBS 114316	*Hordeumvulgare*	Iran	KF144884	KF144928	KF144974	KF145016
*Apiosporaaurea*	CBS 244.83	Air	Spain	AB220251	KF144935	KF144981	KF145023
*Apiosporabalearica*	CBS 145129	Poaceae	Spain	MK014869	MK014836	MK017975	NA
*Apiosporabiserialis*	CGMCC 3.20135	Bamboo	China	MW481708	MW478885	MW522955	MW522938
*Apiosporacamelliae-sinensis*	LC8181	*Brassicacampestris*	China	KY494761	KY494837	KY705229	NA
*Apiosporacamelliae-sinensis*	CGMCC 3.18333	*Camelliasinensis*	China	KY494704	KY494780	KY705173	KY705103
*Apiosporacyclobalanopsidis*	CGMCC 3.20136	*Cyclobalanopsisglauca*	China	MW481713	MW478892	MW522962	MW522945
*Apiosporadescalsii*	CBS 145130	*Ampelodesmosmauritanicus*	Spain	MK014870	MK014837	MK017976	NA
*Apiosporadichotomanthi*	LC8175	*Dichotomanthestristaniiaecarpa*	China	KY494755	KY494831	KY705223	KY705151
*Apiosporadichotomanthi*	CGMCC 3.18332	*Dichotomanthestristaniiaecarpa*	China	KY494697	KY494773	KY705167	KY705096
*Apiosporaesporlensis*	CBS 145136	*Phyllostachysaurea*	Spain	MK014878	MK014845	MK017983	NA
*Apiosporagelatinosa*	GZAAS 20-0107	Bamboo	China	MW481707	MW478889	MW522959	MW522942
*Apiosporaguizhouensis*	LC5318	Air	China	KY494708	KY494784	KY705177	KY705107
*Apiosporaguizhouensis*	CGMCC 3.18334	Air	China	KY494709	KY494785	KY705178	KY705108
*Apiosporahydei*	CBS 114990	Bamboo	China	KF144890	KF144936	KF144982	KF145024
*Apiosporaiberica*	CBS 145137	*Arundodonax*	Portugal	MK014879	MK014846	MK017984	NA
*Apiosporaintestini*	CBS 135835	Gut of a grasshopper	India	KR011352	MH877577	KR011350	NA
*Apiosporaitalica*	CBS 145138	*Arundodonax*	Italy	MK014880	MK014847	MK017985	NA
*Apiosporajiangxiensis*	CGMCC 3.18381	*Maesa* sp.	China	KY494693	KY494769	KY705163	KY705092
*Apiosporakogelbergensis*	CBS 113332	*Cannomoisvirgata*	South Africa	KF144891	KF144937	KF144983	KF145025
*Apiosporakogelbergensis*	CBS 113333	Restionaceae	South Africa	KF144892	KF144938	KF144984	KF145026
*Apiosporamalaysiana*	CBS 102053	*Macarangahullettii*	Malaysia	KF144896	KF144942	KF144988	KF145030
*Apiosporamarii*	CBS 497.90	Air	Spain	AB220252	KF144947	KF144993	KF145035
*Apiosporaneobambusae*	CGMCC 3.18335	Bamboo	China	KY494718	KY494794	KY705186	KY806204
*Apiosporaneobambusae*	LC7107	Bamboo	China	KY494719	KY494795	KY705187	KY705117
*Apiosporaobovata*	CGMCC 3.18331	*Lithocarpus* sp.	China	KY494696	KY494772	KY705166	KY705095
*Apiosporaobovata*	LC8177	*Lithocarpus* sp.	China	KY494757	KY494833	KY705225	KY705153
*Apiosporaovata*	CBS 115042	*Arundinariahindsii*	China	KF144903	KF144950	KF144995	KF145037
*Apiosporaphragmitis*	CBS 135458	*Phragmitesaustralis*	Italy	KF144909	KF144956	KF145001	KF145043
*Apiosporaphyllostachydis*	MFLUCC 18-1101	*Phyllostachysheteroclada*	China	MK351842	MH368077	MK291949	MK340918
*Apiosporapseudoparenchymatica*	CGMCC 3.18336	Bamboo	China	KY494743	KY494819	KY705211	KY705139
*Apiosporapseudospegazzinii*	CBS 102052	*Macarangahullettii*	Malaysia	KF144911	KF144958	KF145002	KF145045
*Apiosporapterosperma*	CBS 134000	*Machaerinasinclairii*	Australia	KF144913	KF144960	KF145004	KF145046
*Apiosporasaccharicola*	CBS 191.73	Air	Netherlands	KF144920	KF144966	KF145009	KF145051
*Apiosporaseptata*	CGMCC 3.20134	Bamboo	China	MW481711	MW478890	MW522960	MW522943
*Apiosporaserenensis*	IMI 326869	NA	Spain	AB220250	AB220344	AB220297	NA
*Apiosporasubrosea*	LC7291	Bamboo	China	KY494751	KY494827	KY705219	KY705147
*Apiosporasubrosea*	CGMCC 3.18337	Bamboo	China	KY494752	KY494828	KY705220	KY705148
*Apiosporaxenocordella*	CBS 595.66	Soil	Austria	KF144926	KF144971	KF145013	KF145055
*Arthriniumcaricicola*	CBS 145127	*Carexericetorum*	Germany	MK014871	MK014838	MK017977	NA
*Arthriniumcrenatum*	CBS 146353	Grass	France	MW208931	MW208861	MW221923	MW221917
*Arthriniumcurvatum*	CBS 145131	*Carex* sp.	Germany	MK014872	MK014839	MK017978	NA
*Arthriniumjaponicum*	IFO 30500	*Carexdespalata*	Japan	AB220262	AB220356	AB220309	NA
*Arthriniumjaponicum*	IFO 31098	*Carexdespalata*	Japan	AB220264	AB220358	AB220311	NA
*Arthriniumluzulae*	AP7619-3	*Luzulasylvatica*	Spain	MW208937	MW208863	MW221925	MW221919
*Arthriniummorthieri*	GZU 345043	*Carexdigitata*	Austria	MW208938	MW208864	MW221926	MW221920
*Arthriniumpuccinioides*	CBS 549.86	*Lepidospermagladiatum*	Germany	AB220253	AB220347	AB220300	NA
*Arthriniumsphaerospermu*m	CBS 146355	Poaceae	Norway	MW208943	MW208865	NA	NA
*Arthriniumsporophleum*	CBS 145154	*Juncus* sp.	Spain	MK014898	MK014865	MK018001	NA
*Bartaliniabella*	CBS 125525	*Maytenusabbottii*	South Africa	GU291796	MH554214	MH554663	MH554421
*Bartaliniapini*	CBS 143891	*Pinuspatula*	Uganda	MH554125	MH554330	MH554797	MH554559
*Beltraniapseudorhombica*	CBS 138003	*Pinustabulaeformis*	China	MH554124	KJ869215	NA	MH554558
*Beltraniarhombica*	CBS 123.58	Sand near mangrove swamp	Mozambique	MH553990	MH554209	MH704631	MH704606
*Beltraniopsisneolitseae*	CPC 22168	*Neolitseaaustraliensis*	Australia	KJ869126	KJ869183	NA	NA
*Broomellavitalbae*	HPC 1154	NA	NA	MH554173	MH554367	MH554846	MH554608
*Castanediellacagnizarii*	CBS 542.96	Leaf litter	Cuba	MH862597	MH874222	NA	NA
*Ciliochorellaphanericola*	MFLUCC 12-0310	Dead leaves	Thailand	KF827444	KF827445	KF827478	KF827477
*Clypeophysalosporalatitans*	CBS 141463	*Eucalyptus* sp.	Portugal	NR_153929	NG_058958	NA	NA
*Clypeosphaeriamamillana*	CBS 140735	*Cornusalba*	France	KT949897	MH554225	MH704637	MH704610
*Cylindriumelongatum*	CBS 115974	*Fagus* sp.	The Netherlands	KM231853	KM231733	KM232123	KM231989
*Diplocerashypericinum*	CBS 109058	*Hypericum* sp.	New Zealand	MH553955	MH554178	MH554614	MH554373
*Disaetaarbuti*	CBS 143903	*Acaciapycnantha*	Australia	MH554148	MH554346	MH554821	MH554583
*Discosiaartocreas*	CBS 124848	*Fagussylvatica*	Germany	MH553994	MH554213	MH554662	MH554420
*Discosiabrasiliensis*	MFLUCC 12-0429	Dead leaf	Thailand	KF827432	KF827436	KF827469	KF827465
*Distononappendiculatabanksiae*	CBS 131308	*Banksiamarginata*	Australia	JQ044422	JQ044442	MH554670	MH554428
*Distononappendiculatacasuarinae*	CBS 143884	*Casuarina* sp.	Australia	MH554093	MH554303	MH554766	MH554527
*Diversimediisporahumicola*	CBS 302.86	Soil	USA	MH554028	MH554247	MH554705	MH554463
*Heterotruncatellaacacigena*	CBS 143880	*Acaciapedina*	Australia	MH554084	MH554295	MH554756	MH554517
*Heterotruncatellaaspera*	CBS 144140	*Acaciaglaucoptera*	Australia	MH554156	MH554352	MH554829	MH554591
*Hyalotiellaspartii*	MFLUCC 13-0397	*Spartiumjunceum*	Italy	KP757756	KP757752	NA	NA
*Hyalotiellatransvalensis*	CBS 303.65	Leaf litter and topsoil of *Acaciakarroo* community	South Africa	MH554029	MH554248	MH554706	MH554464
*Hymenopleellaaustroafricana*	CBS 143886	*Gleditsiatriacanthos*	South Africa	MH554115	MH554320	MH554788	MH554549
*Hymenopleella hippophaëicola*	CBS 113687	*Hippophaë rhamnoides*	Sweden	MH553969	MH554188	MH554628	MH554387
*Immersidiscosiaeucalypti*	NBRC 104195	*Quercusmyrsinifolia*	Japan	AB594790	AB593722	NA	NA
*Lepteutypafuckelii*	CBS 140409	*Tiliacordata*	Belgium	NR_154123	KT949902	MH554677	MH554435
*Lepteutypasambuci*	CBS 131707	*Sambucusnigra*	UK	NR_154124	MH554219	MH704632	MH704612
*Monochaetiamonochaeta*	CBS 115004	*Quercusrobur*	Netherlands	AY853243	MH554198	MH554639	MH554398
*Monochaetiaquercus*	CBS 144034	*Quercuseduardi*	Mexico	MH554171	MH554365	MH554844	MH554606
*Moriniaacaciae*	CBS 137994	*Acaciamelanoxylon*	France	MH554002	MH554221	MH554673	MH554431
*Moriniacrini*	CBS 143888	*Crinumbulbispermum*	South Africa	MH554118	MH554323	MH554791	MH554552
***Neoarthriniumlithocarpicola***	**CFCC 54456**	***Lithocarpusglaber***	**China**	**ON427580**	**ON427582**	**ON456914**	**NA**
***Neoarthriniumlithocarpicola***	**CFCC 55883**	***Lithocarpusglaber***	**China**	**ON427581**	**ON427583**	**ON456915**	**NA**
*Noarthriniummoseri*	CBS 164.80	Dead petiole	Colombia	LN850995	LN851049	LN851154	NA
*Neoarthriniumtrachycarpi*	CFCC 53038	*Trachycarpusfortunei*	China	MK301098	NA	MK303394	MK303396
*Neoarthriniumtrachycarpi*	CFCC 53039	*Trachycarpusfortunei*	China	MK301099	NA	MK303395	MK303397
*Neoarthriniumurticae*	IMI 326344	Leaf litter	India	AB220245	AB220339	NA	NA
*Neopestalotiopsiscubana*	CBS 600.96	Leaf litter	Cuba	KM199347	KM116253	KM199438	KM199521
*Neophysalosporaeucalypti*	CBS 138864	*Corymbiahenryi*	Mozambique	KP004462	MH878627	NA	NA
*Nigrosporaaurantiaca*	CGMCC 3.18130	*Nelumbo* sp.	China	KX986064	KX986098	KY019465	KY019295
*Nigrosporacamelliae-sinensis*	CGMCC 3.18125	*Camelliasinensis*	China	KX985986	KX986103	KY019460	KY019293
*Nigrosporachinensis*	CGMCC 3.18127	*Machilusbreviflora*	China	KX986023	KX986107	KY019462	KY019422
*Nigrosporagorlenkoana*	CBS 480.73	*Vitisvinifera*	Kazakhstan	KX986048	KX986109	KY019456	KY019420
*Nigrosporaguilinensis*	CGMCC 3.18124	*Camelliasinensis*	China	KX985983	KX986113	KY019459	KY019292
*Nigrosporahainanensis*	CGMCC 3.18129	*Musaparadisiaca*	China	KX986091	KX986112	KY019464	KY019415
*Nigrosporalacticolonia*	CGMCC 3.18123	*Camelliasinensis*	China	KX985978	KX986105	KY019458	KY019291
*Nigrosporamusae*	CBS 319.34	*Musa* sp.	Australia	MH855545	KX986110	KY019455	KY019419
*Nigrosporaoryzae*	LC2693	*Neolitsea* sp.	China	KX985944	KX986101	KY019471	KY019299
*Nigrosporaosmanthi*	CGMCC 3.18126	*Osmanthus* sp.	China	KX986010	KX986106	KY019461	KY019421
*Nigrosporapyriformis*	CGMCC 3.18122	*Citrussinensis*	China	KX985940	KX986100	KY019457	KY019290
*Nigrosporarubi*	LC2698	*Rubus* sp.	China	KX985948	KX986102	KY019475	KY019302
*Nigrosporasphaerica*	LC7298	*Nelumbo* sp.	China	KX985937	KX986097	KY019606	KY019401
*Nigrosporavesicularis*	CGMCC 3.18128	*Musaparadisiaca*	China	KX986088	KX986099	KY019463	KY019294
*Nonappendiculataquercina*	CBS 116061	*Quercussuber*	Italy	MH553982	MH554199	MH554641	MH554400
*Parabartalinialateralis*	CBS 399.71	*Acaciakarroo*	South Africa	MH554043	MH554256	MH554719	MH554478
*Parapleurotheciopsisinaequiseptata*	MUCL 41089	Rotten leaf	Brazil	EU040235	EU040235	NA	NA
*Parapleurotheciopsiscaespitosa*	CBS 519.93	*Syzygiumcordatum*	South Africa	MH862437	NG_066263	NA	NA
*Pestalotiopsisadusta*	CBS 263.33	*Rhododendronponticum*	Netherlands	KM199316	KM116198	KM199414	KM199489
*Pestalotiopsisaustralasiae*	CBS 114126	*Knightia* sp.	New Zealand	KM199297	KM116218	KM199409	KM199499
*Phlogicylindriumeucalypti*	CBS 120080	*Eucalyptusglobulus*	Australia	NR_132813	DQ923534	MH704633	MH704607
*Phlogicylindriumeucalyptorum*	CBS 120221	*Eucalyptusglobus*	Australia	EU040223	MH554204	MH704635	MH704608
*Pseudopestalotiopsisampullacea*	LC6618	*Camelliasinensis*	China	KX895025	KX895039	KX895358	KX895244
*Pseudopestalotiopsiscamelliae-sinensis*	LC3009	*Camelliasinensis*	China	KX894935	KX895050	KX895267	KX895152
*Pseudosarcostromaosyridicola*	CBS 103.76	*Osyrisalba*	France	MH553954	MH554177	MH554613	MH554372
*Pseudosporidesmiumknawiae*	CBS 123529	NA	NA	MH863299	MH874823	NA	NA
*Robillardaafricana*	CBS 122.75	NA	South Africa	KR873253	KR873281	MH554656	MH554414
*Robillardaterrae*	CBS 587.71	Soil	India	KJ710484	KJ710459	MH554734	MH554493
*Sarcostromaafricanum*	CBS 143879	*Pelargoniumcucullatum*	South Africa	MH554078	MH554289	MH554752	MH554513
*Sarcostromaaustraliense*	CBS 144160	*Daviesialatifolia*	Australia	MH554138	MH554340	MH554811	MH554573
*Seimatosporiumgermanicum*	CBS 437.87	NA	Germany	MH554047	MH554259	MH554723	MH554482
*Seimatosporiumluteosporum*	CBS 142599	*Vitisvinifera*	USA	KY706284	KY706309	KY706259	KY706334
*Seiridiumcancrinum*	CBS 226.55	*Cupressusmacrocarpa*	Kenya	LT853089	MH554241	LT853236	LT853186
*Seiridiumcupressi*	CBS 224.55	*Cupressusmacrocarpa*	Kenya	LT853083	MH554240	LT853230	LT853180
*Sporocadusbiseptatus*	CBS 110324	NA	NA	MH553956	MH554179	MH554615	MH554374
*Sporocaduscornicola*	CBS 143889	*Cornussanguinea*	Germany	MH554121	MH554326	MH554794	MH554555
*Sporocadustrimorphus*	CBS 114203	*Rosacanina*	Sweden	MH553977	MH554196	MH554636	MH554395
*Strickeriakochii*	CBS 140411	*Robiniapseudoacacia*	Austria	NR_154423	KT949918	MH554679	MH554437
*Subramaniomycesfusisaprophyticus*	CBS 418.95	Leaf litter	Cuba	EU040241	EU040241	NA	NA
*Synnemapestaloidesjuniperi*	CBS 477.77	*Juniperusphoenicea*	France	MH554053	MH554266	MH554729	MH554488
*Truncatellaangustata*	CBS 113.11	*Piceaabies*	Germany	MH553966	MH554185	MH554625	MH554384
*Xenoseimatosporiumquercinum*	CBS 129171	*Rhododendron* sp.	Latvia	MH553997	MH554216	MH554666	MH554424
*Xyladictyochaetalusitanica*	CBS 143502	*Eucalyptus* sp.	Australia	MH107926	MH107972	MH108053	MH108033

Note: NA, not applicable. Strains in this study are marked in bold.

## ﻿Results

### ﻿Phylogenetic analyses

**Figure 1.**
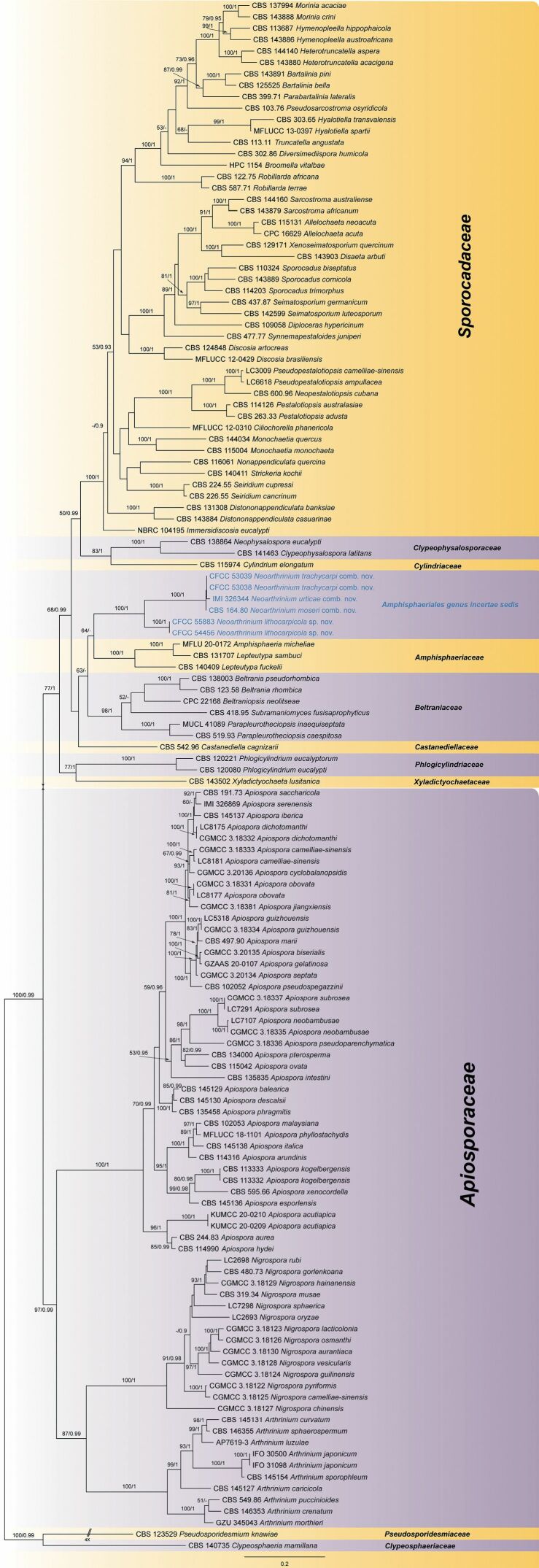
Phylogram of Amphisphaeriales resulting from a Maximum Likelihood analysis, based on a combined matrix of ITS, LSU, *tef1* and *tub2*. Numbers above the branches indicate ML bootstraps (left, ML BS ≥ 50%) and Bayesian Posterior Probabilities (right, BPP ≥ 0.90). The tree is rooted with *Clypeosphaeriamamillana* (CBS 140735) and *Pseudosporidesmiumknawiae* (CBS 123529). New species and combinations proposed in the present study are marked in blue.

The combined sequence dataset (ITS, LSU, *tef1* and *tub2*) was analysed to infer the phylogenetic placement of our new isolates within Amphisphaeriales. The dataset consisted of 136 sequences, including two outgroup taxa, *Clypeosphaeriamamillana* (CBS 140735) and *Pseudosporidesmiumknawiae* (CBS 123529). A total of 3526 characters, including gaps (793 for ITS, 859 for LSU, 762 for *tef1* and 1112 for *tub2*), were included in the phylogenetic analysis. Of these characters, 1543 were constant, 284 were variable, but parsimony-uninformative and 1699 were parsimony-informative. The best ML tree (lnL = - 72640.48) revealed by RAxML is shown in Fig. 1. The topologies resulting from ML and BI analyses of the concatenated dataset were congruent (Fig. 1). Isolates CFCC 54456 and CFCC 55883 from the present study, together with CFCC 53038, CFCC 53039, CBS 164.80 and IMI 326344, formed a clade distinct from Apiosporaceae and the other families in Amphisphaeriales. Hence, a new genus named *Neoarthrinium* is proposed herein for this clade. *Arthriniumtrachycarpi*, *Ar.urticae* and *Wardomycesmoseri* are transferred to *Neoarthrinium*. In addition, the two new isolates (CFCC 54456 and CFCC 55883) that form a sister clade to *N.moseri*, *N.trachycarpi* and *N.urticae* are described here as the new species *N.lithocarpicola*.

### ﻿Taxonomy

#### ﻿ Neoarthrinium

Taxon classificationFungiAmphisphaerialesApiosporaceae

Ning Jiang gen. nov.

0F22D7F7-D920-516B-90DB-F1A07FFDFA3C

843845

##### Etymology.

Named after its morphological similarity to *Arthrinium*.

##### Type species.

*Neoarthriniumlithocarpicola* Ning Jiang

##### Description.

Hyphae formed on PDA hyaline, branched, septate. Asexual morph: Conidiophores cylindrical, septate, verrucose, flexuous, sometimes reduced to conidiogenous cells. Conidiogenous cells erect, blastic, aggregated in clusters on hyphae, hyaline to pale brown, smooth, doliiform, subglobose to lageniform, branched. Conidia brown to dark brown, smooth to finely roughened, subglobose, ellipsoid to lenticular, with a longitudinal germ slit, occasionally elongated to ellipsoidal. Sexual morph: Undetermined.

#### ﻿ Neoarthrinium lithocarpicola

Taxon classificationFungiAmphisphaerialesApiosporaceae

Ning Jiang sp. nov.

080D8973-BDAF-5A96-AB80-82D4041D2664

843846

Fig. 2

##### Etymology.

Named for its host genus “*Lithocarpus*” and “-*cola*” = inhabiting.

##### Description.

Hyphae 1.5–4.5 μm diam., hyaline, branched, septate. Asexual morph: Conidiophores cylindrical, septate, verrucose, flexuous, sometimes reduced to conidiogenous cells. Conidiogenous cells erect, blastic, aggregated in clusters on hyphae, hyaline to pale brown, smooth, globose to subglobose, branched, (4–)5.5–8 × 2.5–3.5(–4) μm, mean ± SD = 6.6 ± 1.3 × 3.1 ± 0.4 μm, n = 50. Conidia brown to dark brown, smooth to finely roughened, subglobose to lenticular, with a longitudinal germ slit, occasionally elongated to ellipsoidal, (5–)6–8(–8.5) × (4.5–)5–5.5(–6) μm, mean ± SD = 7 ± 0.8 × 5.3 ± 0.5 μm, L/W = 1.1–1.8, n = 50. Sexual morph: Undetermined.

**Figure 2.**
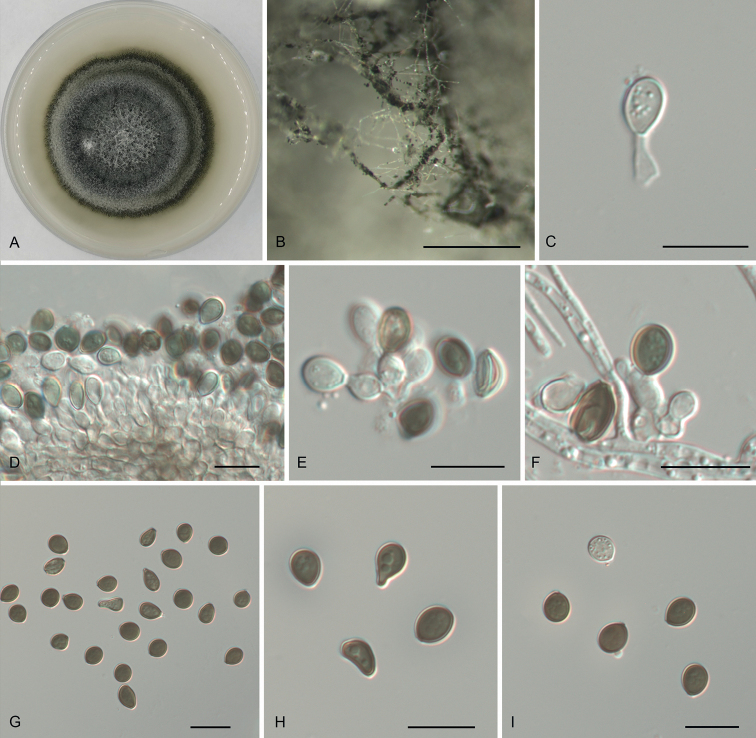
*Neoarthriniumlithocarpicola***A** colony on PDA**B** conidiomata formed in culture **C–F** conidiogenous cells giving rise to conidia **G–I** conidia. Scale bars: 500 μm (**B**), 10 μm (**C–I**).

##### Culture characters.

Colonies on PDA flat, spreading, with flocculent aerial mycelium forming concentric rings, edge entire, mouse grey to greyish-green, reaching 60 mm diam. after 10 d at 25 °C, forming abundant conidiomata.

##### Specimens examined.

China. Guangdong Province, Guangzhou City, on leaf spots of *Lithocarpusglaber* (Thunb.) Nakai, *Shang Sun* (holotype CAF800050 = JNH0046; ex-type living culture: CFCC 54456; other living culture: CFCC 55883).

##### Notes.

Two isolates of *Neoarthriniumlithocarpicola* from *Lithocarpusglaber* (Thunb.) Nakai formed a well-supported monophyletic clade, distinct from *N.moseri*, *N.trachycarpi* and *N.urticae* (Fig. 1). Morphologically, *N.lithocarpicola* is distinguished from *N.moseri* in smaller conidia (5–8.5 × 4.5–6 µm in *N.lithocarpicola* vs. 10–14 × 3–4.5 µm in *N.moseri*; Gams 1995). *Neoarthriniumlithocarpicola* is different from *N.urticae* by lacking thick blackish septa in conidiophores (Ellis 1965). *Neoarthriniumlithocarpicola* is similar to *N.trachycarpi* in the size of its conidiogenous cells and conidia, but it can be distinguished by its globose to subglobose conidiogenous cells (Yan et al. 2019).

#### ﻿ Neoarthrinium moseri

Taxon classificationFungiAmphisphaerialesApiosporaceae

(W. Gams) Voglmayr comb. nov.

F199C77A-6306-51CB-A79A-84705D6FABDA

844772

##### Basionym.

*Wardomycesmoseri* W. Gams, Beih. Sydowia 10: 67 (1995)

##### Notes.

Based on a placement within Xylariales in phylogenetic analyses, Sandoval-Denis et al. (2016) excluded this species from the genus (Microascales); however, they did not suggest an alternative generic classification. The blastic hyaline, smooth, lageniform conidiogenous cells aggregated in clusters and the subglobose to ellipsoid dark brown conidia with a longitudinal germ slit (Gams 1995) fully matched the genus *Neoarthrinum*. The ITS, LSU and *tub2* sequences of the ex-holotype strain of *N.moseri* (CBS 164.80) are almost identical to those of *N.trachycarpi*, indicating that they may be synonymous. Both species were isolated from petioles of palms: *N.moseri* from *Mauritiaminor* Burret in Colombia and *N.trachycarpi* from *Trachycarpusfortunei* (Hook.) H.Wendl. in China. However, the two species were reported to differ in conidial size (10–14 × 3–4.5 µm in *N.moseri* vs. 6.1–8.5 × 4.2–5.8 μm in *N.trachycarpi*; Gams 1995; Yan et al. 2019) and for the time being, we therefore kept them separate.

#### ﻿ Neoarthrinium trachycarpi

Taxon classificationFungiAmphisphaerialesApiosporaceae

(C.M. Tian & H. Yan) Ning Jiang comb. nov.

FDA9EAE0-58E6-5223-B458-E47E1D6B83C2

843847

##### Basionym.

*Arthriniumtrachycarpi* C.M. Tian & H. Yan [as ‘*trachycarpum*’], Phytotaxa 400(3): 208 (2019)

#### ﻿ Neoarthrinium urticae

Taxon classificationFungiAmphisphaerialesApiosporaceae

(M.B. Ellis) Ning Jiang comb. nov.

AC635989-1929-5909-8FE9-29C2E571041F

843848

##### Basionym.

*Arthriniumurticae* M.B. Ellis, Mycol. Pap. 103: 16 (1965)

##### Notes.

The possibility that *Apiosporellaurticae* (Rehm) Höhn. is the sexual morph of *Arthriniumurticae* is raised by the fact that both share the same host (*Urtica*) and are classified as members of the Apiosporaceae (Index Fungorum, accessed 4 July 2022). This evidence would have far reaching nomenclatural consequences not only for species, but also for generic classification, as *Apiosporella* (Höhnel 1909) may then qualify for an older genus name to be used for *Neoarthrinium*. However, according to L. Holm, the holotype specimen of its basionym, *Apiosporaurticae* (S-F12119), represents a very different fungus, *Didymellaeupyrena* (Didymellaceae, Pleosporales, Dothideomycetes; https://herbarium.nrm.se/specimens/F12119, accessed 4 July 2022). The status of the genus *Apiosporella* is still unclear because Höhnel (1909) did not choose a type from the six different species included in the genus. However, none of the original species is a close relative of Apiosporaceae or *Neoarthrinium*; therefore, *Apiosporella* should be excluded from Apiosporaceae.

No sequence data are available for isolates from the type host *Urticadioica* L. (Urticaceae). The single culture sequenced (IMI 326344) was isolated from unidentified leaf litter collected in India. Additional molecular studies on verified isolates from *Urtica* collected in Europe are necessary to reveal whether IMI 326344 represents true *N.urticae*. However, *N.urticae* appears to be very rare and we are unaware of any additional collections with the exception of the type.

## ﻿Discussion

*Arthrinium* and related genera are important fungal taxa whose concepts and classification have undergone many changes and additions (e.g. Cooke 1954; Samuels et al. 1981; Larrondo and Calvo 1990; Hyde et al. 1998; Jaklitsch and Voglmayr 2012; Crous and Groenewald 2013; Singh et al. 2013; Sharma et al. 2014; Dai et al. 2016, 2017; Hyde et al. 2016; Jiang et al. 2018, 2020; Wang et al. 2018; Pintos et al. 2019; Pintos and Alvarado 2021). In recent years, substantial changes in classification were implemented in the course of unitary nomenclature. A large number of newly-discovered species have been described as a result of extensive sampling of new isolates, based on multigene phylogenies (e.g. Crous and Groenewald 2013; Wang et al. 2018; Pintos and Alvarado 2021). Currently, *Arthrinium*-like asexual morphs are shared by three distinct lineages within Amphisphaeriales, viz. *Apiospora*, *Arthriniums. s.* and *Neoarthrinium* as shown in Fig. 1. *Arthriniums. s.* is the sister genus to *Nigrospora*, which morphologically differs from *Apiospora*, *Arthrinium* and *Neoarthrinium* in conidial ontogeny (Wang et al. 2017). The phylogram shown in Fig. 1 is consistent with that shown in Tian et al. (2021) in placing *Apiospora*, *Arthrinium* and *Nigrospora* within a clade that is distinct from the new genus *Neoarthrinium*, although *Apiospora* and *Arthrinium* share conidial morphology similar to that of *Neoarthrinium*.

Morphologically, *Apiospora*, *Arthrinium* and *Neoarthrinium* are similar in having basauxic conidiogenesis. Conidia of *Apiospora* and *Neoarthrinium* are generally more or less rounded in face view and lenticular in side view, while those of *Arthrinium* are variously shaped, viz. globose, angular, polygonal, curved, fusiform or navicular (Yan et al. 2019; Pintos and Alvarado 2021). However, the conidiophores of several *Arthrinium* and *Neoarthrinium* species have thick blackish septa, which are rarely observed in *Apiospora* (Ellis 1965; Wang et al. 2018; Pintos and Alvarado 2021). Hence, these three genera are difficult to distinguish by only asexual morphology.

Regarding their hosts, there are some tendencies in host preferences, while *Arthrinium* species are predominantly found in Cyperaceae and Juncaceae (Pintos and Alvarado 2021) and species of *Apiospora* primarily occur on Poaceae (but also on many other hosts; Wang et al. 2018). Four *Neoarthrinium* species were discovered on four hosts from three distantly-related host families (i.e. *N.lithocarpicola* from *Lithocarpusglaber* (Thunb.) Nakai, Fagaceae; *N.moseri* from *Mauritiaminor* Burret, Arecaceae; *N.trachycarpi* from *Trachycarpusfortune* (Hook.) H.Wendl., Arecaceae; and *N.urticae* from *Urticadioica* L., Urticaceae; Ellis 1965; Yan et al. 2019). Hence, host association is not a fully reliable feature to distinguish *Apiospora*, *Arthrinium* and *Neoarthrinium*.

Compared to species, generic delimitation is much more subjective. However, there is a broad agreement that genera, along with all taxonomic classification units at all ranks, should be monophyletic. As morphology is frequently insufficient for phylogenetic classification, molecular evidence is regarded as significant data or even an essential characteristic in the classification and identification of fungal taxa. In the present study, *Neoarthrinium* is proposed as a new genus for a group of species phylogenetically distinct from *Apiospora*, *Arthrinium* and *Nigrospora* to maintain monophyletic *Arthrinium*-like genera. Using morphological and phylogenetic data, however, we need more samples to improve our understanding of *Arthrinium*-like taxa and genera in the Amphisphaeriales.

## Supplementary Material

XML Treatment for Neoarthrinium

XML Treatment for Neoarthrinium lithocarpicola

XML Treatment for Neoarthrinium moseri

XML Treatment for Neoarthrinium trachycarpi

XML Treatment for Neoarthrinium urticae
